# P-1449. Communication Strategies to Increase HPV Vaccination Intention

**DOI:** 10.1093/ofid/ofaf695.1635

**Published:** 2026-01-11

**Authors:** Florence Momplaisir, John Jemmott, Aasith Villavicencio Paz, Sukyung Kim, Tara McWilliams, Qufei Wu, Hervette Nkwihoreze, Jessica Fishman

**Affiliations:** University of Pennsylvania, Villanova, PA; University of Pennsylvania, Villanova, PA; Hospital of the University of Pennsylvania, Philaldelphia, Pennsylvania; Thomas Jefferson University, Philadelphia, Pennsylvania; University of Pennsylvania, Villanova, PA; University of Pennsylvania, Villanova, PA; University of Pennsylvanis, Philadelphia, Pennsylvania; University of Pennsylvania, Villanova, PA

## Abstract

**Background:**

The HPV vaccine is highly effective at reducing cervical cancer and other HPV-related cancers. Vaccination is recommended among adults between ages 18-45 but uptake has been low. Among a national sample of adults in this age range, we conducted an online experiment to identify the types of messages that can increase HPV vaccination intentions.

**Figure:**
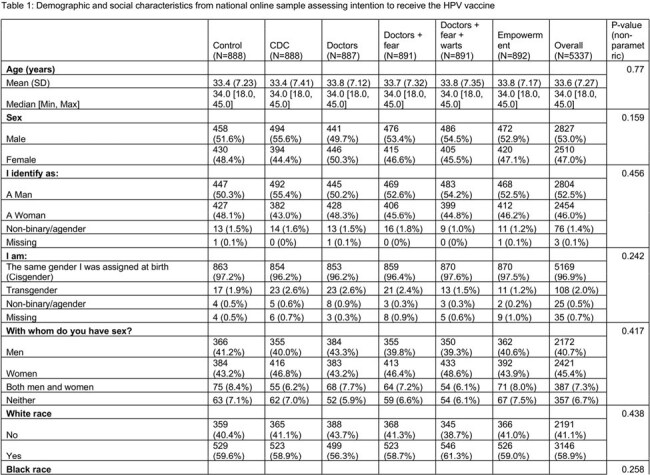

**Figure:**
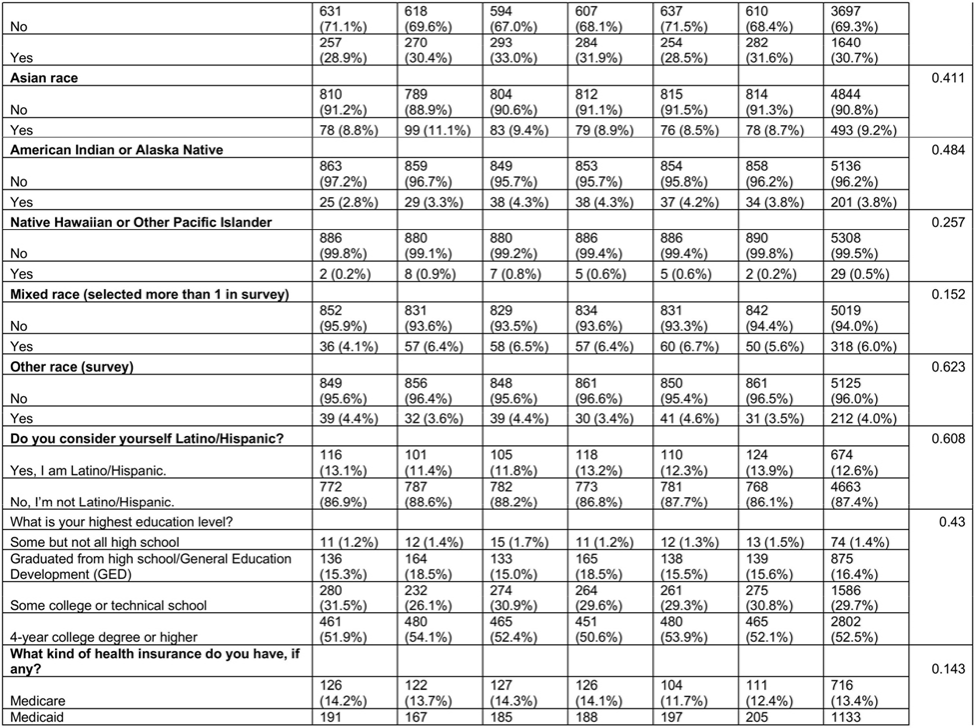

**Methods:**

We used Prolific, an online platform to enroll a national sample of adults ages 18-45 who had not received the HPV vaccine. The primary outcome was a validated 3-item scale assessing HPV vaccination intention. We randomly assigned participants in equal numbers to one of five theory-based HPV vaccine message conditions or an attention-matched control arm. To compare the outcome between intervention groups and the control group, we used the Tukey’s HSD to account for multiple comparisons. Using a linear regression model with planned contrasts, we tested the efficacy of each experimental arm compared with the attention-control condition in increasing vaccination intention adjusting for demographic and social variables.

**Figure:**
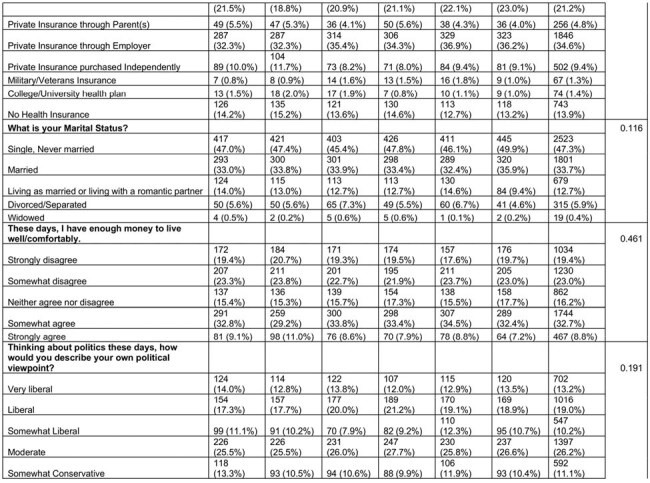

**Figure:**
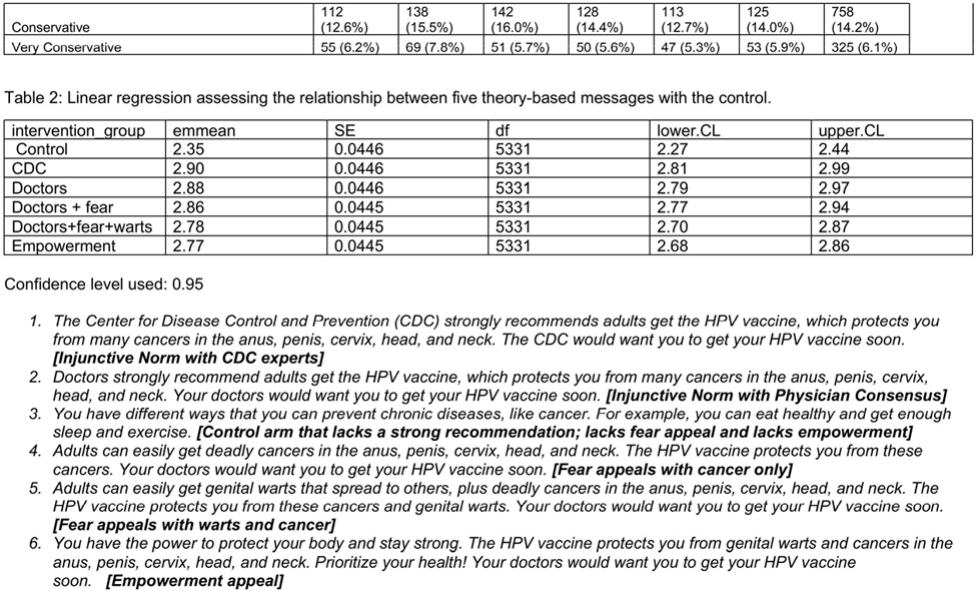

**Results:**

Between March and April 2005, we enrolled 5337 participants, 47% female and 30% Black (Table 1). Each theory-based message performed better than the control but there were no significant differences between the intervention messages. However, in the linear regression (Table 2), we found that messages with the strongest effects communicated injunctive norms from Center for Disease Control and Prevention experts with an estimated marginal mean of 2.90 (95% CI 2.81-2.99), followed by injunctive norm with physician consensus (EMM 2.88, 95% CI 2.79-2.97), and a fear appeal from HPV-related cancer (EMM 2.86, 95% CI 2.77-2.94). The empowerment-based message had the weakest performance (estimated marginal mean 2.77 (95% CI 2.68-2.86). While demographic co-variates differed by vaccine intention, they did not affect the intervention efficacy in our linear regression.

**Conclusion:**

Using a large national sample, we were able to identify theory-based messages that increased intention to receive the HPV vaccine. Our findings may inform public health messaging to increase HPV vaccination.

**Disclosures:**

All Authors: No reported disclosures

